# Scientia pro bono humani generis: Science for the benefit of humanity

**DOI:** 10.1017/cts.2023.696

**Published:** 2024-02-20

**Authors:** Laura P. James, Robert Kimberly, Christopher John Lindsell, Jareen K. Meinzen-Derr, Ruth O’Hara

**Affiliations:** 1 University of Arkansas for Medical Sciences (UAMS) and Arkansas Children’s Hospital, Little Rock, AR, USA; 2 University of Alabama at Birmingham Heersink School of Medicine, Birmingham, AL, USA; 3 Data Science and Biostatistics, Duke University, Duke Clinical Research Institute, Durham, NC, USA; 4 Division of Biostatistics and Epidemiology, Cincinnati Children’s Hospital Medical Center, Center for Clinical and Translational Science and Training, University of Cincinnati College of Medicine, Cincinnati, OH, USA; 5 Department of Psychiatry and Behavioral Sciences, Stanford University School of Medicine, Stanford, CA, USA

Clinical trials have been defined by the World Health Organization as a type of research that investigates new tests and treatments and evaluates their effects on human health outcomes [1]. This definition underpins the sentiments of Zarin and Goodman expressed in a 2019 commentary entitled “Harms from uninformative trials” [2]. The authors defined an *uninformative trial* as one lacking in meaning by the patient, clinician, researcher, or policymaker. A lack of meaningful use is characteristic of a large number of trials developed during the COVID-19 pandemic; only 5% were randomized or had sufficient power to provide meaningful clinical data [3]. Academic health organizations, funding agencies, and clinical trialists have been challenged to optimize clinical trial informativeness, and quality issues continue to plague the development and conduct of clinical trials. Numerous factors may influence clinical trial informativeness, including the complexity of the study design, local site issues that compromise trial conduct, and the lack of inclusion of racial and ethnic minority populations, which limits the generalizability of trial results. Informativeness drives the likelihood that clinical trial results will influence clinical practice and improve health outcomes.

This issue of *The Journal of Clinical and Translational Science* highlights innovations for enhancing the informativeness and quality of clinical trials. Manuscripts address one of the following topics: (1) local clinical site infrastructure and readiness, including educational programs, (2) multisite clinical trial site planning, (3) perspectives on new clinical trial designs, and (4) strategies for optimizing the recruitment and retention of racial and ethnic minority populations into clinical trials.

## Local Site Infrastructure and Readiness

Kost *et al*. [4] detail the experiences of Rockefeller University in building a clinical and translational science program guided by navigation of a senior core of clinical trial experts and other programs established to enhance clinical trial quality and rigor, building upon the sentiment *Scientia pro bono humani generis* (Science for the benefit of humanity). Buse *et al.* [5] report on a multiyear project involving diverse clinical trialists with representation from the private sector (Flagship Pioneering, Medable, Inc.), a healthcare organization (Harbor Health), the National Academies of Sciences, Engineering, and Medicine, and the National Center for Advancing Translational Sciences. Their effort is to develop a framework for clinical trial site readiness based on existing trial site qualifications from industry sponsors across six domains: research team, infrastructure, study management, data collection and management, quality oversight, and ethics and safety. Implementation of the framework is expected to reduce inefficiencies, serve as guidance to new sites wishing to enter the clinical trial enterprise, and increase engagement with underrepresented communities [6]. Institutional infrastructure investments, such as the adoption and implementation of a robust clinical trial management system, can provide an approach to monitor enterprise-wide metrics for clinical trial operations, enabling the identification of factors within the institution that contribute to inefficiencies. The experiences of Duke University implementing the OnCore clinical research management system are reported by Mullen *et al.* [7].

## Multisite Clinical Trials

Lane *et al*. [8] offer guidance for multisite clinical trial planning, reflecting experiences of the Trial Innovation Network (TIN) of the National Center for Advancing Translational Sciences (NCATS). TIN was expressly informed by NCATS to increase multisite trial efficiency and effectiveness. As highlighted by Lane *et al*. [8], the transition from single-center trials to multicenter trials is complex and “controlling conditions that support feasibility, trial conduct, and trial reporting becomes harder as research is scaled-up across diverse centers.” However, multisite trials have the ability to be more informative than single-site trials as they provide greater evidence on the generalizability of an intervention, provide external validation of the protocol, support equipoise in more than one clinical setting, and identify design and operational details that can positively influence trial informativeness. Three principles are offered to support the informativeness and quality of multisite trials: (1) assemble a diverse team, (2) leverage existing processes and systems, and (3) carefully consider budget and contract factors that may prevent informativeness.

## New Clinical Trials Designs

Adaptive clinical trial designs can enhance the informativeness of clinical trials. Adaptive designs can enhance efficiency and the potential to enroll a smaller number of subjects, sparing resources [9]. Because adaptive designs incorporate results accumulating during the trial to modify the course of the trials (using prespecified rules), they are purposed to be more informative than traditional trial designs given the same trial resources. Kaiser *et al*. review innovations in adaptive trial designs, focusing on the seven major elements of adaptation described in the 2019 Food and Drug Administration guidance; these authors offer relevant examples of these design elements [10]. Roddy *et al*. provide a state-of-the-art review of adaptive step-wedge clinical trials that used behavior change-oriented interventions in the management of chronic disease [11]. The 22 studies in the review included singly randomized trials (SRTs) which are traditional designs where participants are randomized only once, and also sequential multiple assignment randomized trials (SMARTs), where participants are randomized at each sequential stage of treatment, informed by prior treatment response [11]. SMARTs test adaptive interventions systematically and efficiently, saving resources and asking multiple questions about components of an adaptive intervention in a high-quality manner without unduly increasing sample size [11]. The review also identifies future directions for subsequent trials, such as the need to understand what behavioral adaptations are needed, and for whom [11].

## Engagement with Underrepresented Populations

Recruitment of underrepresented research participants continues to be a major challenge for all clinical trials, hindering the broad uptake of clinical innovations that can improve health outcomes. Hefferman *et al*. [12] report on the perspectives of clinical research coordinators (CRCs) regarding perceived barriers and facilitators to the recruitment of underrepresented research participants. CRCs viewed the translation of study documents for non-English-speaking potential participants as resulting in participants being much more willing (27%) or somewhat more willing (37%) to participate in clinical trials. Participation in cultural competency training had a marginal effect on the confidence of CRCs approaching individuals of a different background about research participation; 43% of CRCs reported that the training made no difference and 36% reported that it made them less confident [12]. This study highlights the need for additional research on effective training strategies for CRC’s and other research staff.

The experience gained by investigators conducting research for many years in the remote areas of Africa, rural China, and urban South Asia has direct application to expanding the inclusion of underrepresented populations in the USA. Fisher-Hock *et al*. [13] share their experiences with research participants who may otherwise be excluded from clinical research because of cultural, economic, linguistic, or geographic reasons. Keys to successful engagement with underrepresented populations included (1) gaining a deep understanding of the community, (2) co-development of the study with the community, and (3) implementation of community-led study recruitment and community-informed study procedures.

Embracing technological approaches for analysis, a machine learning approach to identify factors associated with recruitment success identified participant compensation and the trial funding source as the two most important features informing recruitment rates among the 30 evaluated in this review of 393 randomized controlled trials (RCTs) [14]). Government-funded RCTs were more likely to be successful, while industry-funded studies were less likely to be successful. Shorter protocols, more likely associated with industry studies, had lower recruitment success, perhaps due to the faster pace and greater constraints of industry sponsored RCT [14]. Research participant compensation has been identified in previous studies and underscores the importance of compensation in motivating potential research participants [14]. The authors emphasize the need for “ethical vigilance” in determining appropriate participant compensation, emphasizing the need to guard against financial exploitation [14]. Moreover, they advise that there is no “one-fitting-all” approach for recruitment, calling for flexible infrastructure for research participant recruitment.

All of the efforts highlighted in this issue underscore that multiple strategies are needed and are being explored for ensuring the informativeness of clinical trials. As academic health centers, study sponsors, and translational researchers and staff seek to optimize the clinical trials enterprise, it is critical to maintain at the center the need for trials to be informative. To do otherwise would dismiss and waste the opportunity and privilege of leveraging science to benefit humanity.
